# A comparative analysis of traditional and technology-supported stepped teaching for clock and fraction reading skills in students with dyscalculia

**DOI:** 10.3389/fpsyg.2026.1850481

**Published:** 2026-06-04

**Authors:** Nurcan Kaya, Gönül Akçamete, Mukaddes Sakallı Demirok

**Affiliations:** Faculty of Education, Near East University, Lefkoşa, Cyprus

**Keywords:** clock teaching, fraction teaching, mathematical learning difficulty (dyscalculia), stepped teaching method, web application

## Abstract

This study aimed to compare the effectiveness of technology-supported stepped teaching method in the acquisition of clock reading/telling and fraction reading/telling skills among students with mathematics learning difficulties. Six students from a special education school in the Turkish Republic of Northern Cyprus participated in the study. A single-subject adapted alternating treatments design was employed, and baseline, instructional, daily probe, maintenance, and generalization sessions were conducted in a one-to-one instructional format. Clock and fraction instruction incorporated analog clocks, fraction models, illustrated cards, and a tablet-based AI application, and was structured according to the instructional steps of “do,” “show,” and “say”. Student responses were systematically recorded, including correct, incorrect, and no responses, as well as the levels of verbal and physical prompting required. Maintenance and generalization sessions were conducted to assess the durability of acquired skills and their generalization across different materials, settings, and instructors. The findings indicated that both instructional approaches were effective in improving students’ clock reading/telling and fraction reading/telling skills.

## 1 Introduction

Mathematics difficulty (dyscalculia) is addressed within the subcategory of “mathematical impairment” under specific learning disorders, according to the Diagnostic and Statistical Manual of Mental Disorders, Fifth Edition (DSM-5), published by the American Psychiatric Association (2013). DSM-5 defines it as a condition persisting for at least 6 months despite targeted and evidence-based interventions, characterized by difficulties in understanding numerical concepts, comprehending quantitative relationships, and performing arithmetic operations accurately and fluently, along with persistent deficits in calculation and mathematical reasoning skills.

Dyscalculia, which forms the basis of this study, is a specific learning difficulty in mathematics that negatively affects the acquisition and application of arithmetic skills and has genetic, neurobiological, and epidemiological underpinnings (Shalev, 2004). This difficulty is not solely determined by biological factors but is also influenced by inadequate instruction, socioeconomic conditions, and environmental factors. Epidemiological studies report that dyscalculia affects approximately 3–6% of children (Kucian and Von Aster, 2015). Difficulties in numerical computation can impede children’s academic achievement and adversely affect problem solving, time management, and use of money—critical skills for daily life. Thus, dyscalculia extends beyond academic performance, representing a multifactorial, developmental learning problem that impacts an individual’s functional competence.

Acquiring academic skills is essential for students’ success in daily life and for maintaining independence (Erbaş, 2008). In addition to reading and writing, mathematical skills such as telling time, using money, understanding fractions, performing arithmetic calculations, and problem solving are critical for effective participation in everyday activities (Snell and Brown, 2006). Consequently, teaching these skills to students with dyscalculia is crucial, not only for academic achievement but also for fostering independent living, social adaptation, and overall quality of life. Instruction should be planned using individualized and application-oriented strategies (Butterworth, 2005; Geary, 2011; MEB, 2021).

Errorless learning, direct instruction, and the point determination technique are among approaches recognized as effective in the teaching of mathematical skills and concepts (Gürsel, 2017; Yıkmış et al., 2018). Within these approaches, the stepped teaching method is particularly notable for its systematic and structured design. The method delineates teacher-student-material interactions as input–output features, encompassing 16 different combinations, which facilitates student learning through a progression from concrete to abstract concepts (Gürsel, 2017).

Numerous studies have documented the use of the stepped teaching method for teaching various mathematical skills. Examples include addition and subtraction (Balçık, 2015; Dağseven, 2001; Gınalı-Göriş, 2006; Şafak, 2007; Yıkmış, 1999; Gersten et al., 2009; Montague, 2008; Kitchens, 2012), use of money (Montague, 2008; Yıkmış et al., 2006), and concept acquisition (Sazak Pınar, 2013; Varol, 2009; Bryant et al., 2000; Fuchs et al., 2008). Similarly, clock-reading and fraction-understanding skills are critical both for daily life and within educational programs; however, research on the application of the stepped teaching method for these skills remains limited.

Burny et al. (2012) emphasized that students with special needs experience significant difficulties in learning to read clocks, and highlighted clock instruction as a complex yet essential skill that can be effectively taught using structured methods. Likewise, Van De Pol et al. (2010) reported that stepped teaching strategies enhance student independence and promote durable learning. However, literature shows that studies employing the stepped teaching method specifically for teaching clock-reading and fraction skills to students with learning disabilities or dyscalculia are scarce. Existing studies since the 2000s have largely focused on populations with intellectual disabilities, autism, or hearing impairments, indicating a notable gap in research on students with specific learning difficulties. Furthermore, when considering publications after 2018, technology-supported applications of the stepped teaching method in teaching clock- and fraction-reading skills are quite rare.

From a theoretical perspective, these findings can be interpreted through cognitive load theory and information processing theory. Students with dyscalculia often experience increased cognitive load due to working memory limitations and difficulties in multi-step mathematical tasks. In this context, instructional design is important for supporting efficient learning and reducing unnecessary cognitive demands. The technology-supported format may reduce extraneous cognitive load by providing structured visual supports, step-by-step guidance, instant feedback, and adaptive prompting. These features can help learners identify and correct errors more quickly and support more efficient information processing compared to traditional instruction, where feedback is typically less immediate.

Today, tablet computers and technology-based instructional systems, particularly when integrated with the stepped teaching method, make significant contributions to the development of mathematical and academic skills among students with special educational needs. Tablet applications have been shown to support the understanding of reading content, number–object correspondence, and functional mathematical skills, with international research reporting consistently positive outcomes (Özbek, 2019; Öztürk, 2016; Eliçin et al., 2015; Acungil, 2014; Hammond et al., 2010; Kagohara et al., 2011; Allen et al., 2012; Burke et al., 2013; Cannella-Malone et al., 2013; Doenyas et al., 2014).

Technology-based systems analyze students’ performance data, enabling individualized instruction through the adjustment of teaching steps and prompt levels. When combined with the stepped teaching method, these systems support data-driven decision-making and provide immediate feedback, thereby facilitating personalized instruction (Cannella-Malone et al., 2013). Such findings suggest that the integration of stepped teaching and technology-based approaches may offer an effective and sustainable strategy for the acquisition of mathematical skills by students with special educational needs.

Technology-supported interventions have also demonstrated effectiveness in teaching clock-reading and fraction-reading skills to students with special needs. For example, Bouck and Hunley (2014) implemented an iPad-based application for students with intellectual disabilities in clock-telling instruction, yielding significant improvements. Similarly, Mechling (2011) reported positive outcomes using video modeling and technology-assisted strategies for understanding and expressing time.

Mathematical skills such as clock reading and fraction understanding are essential for performing basic daily life tasks. For individuals with special educational needs, these skills constitute an integral component of independent living. This highlights both the current practical importance and the gaps in the literature. For students with learning difficulties, acquiring clock- and fraction-reading skills in daily life is critically important, and comparative evaluations of different instructional presentations—particularly technology-supported approaches—are clearly needed. Addressing this need, the present study applies the stepped teaching method with different presentations to clock-telling/reading and fraction-reading/telling skills, forming the central focus of the research.

The aim of this study is to compare the effectiveness and efficiency of traditional and technology -supported presentations of the stepped teaching method in teaching clock- and fraction-reading/telling skills to students with dyscalculia. Specifically, the study examines the effectiveness of both instructional models in terms of acquisition, monitoring, and generalization of clock- and fraction-reading skills, as well as differences in the number of sessions and trials, number of incorrect responses, and total instructional time—all being considered as indicators of instructional productivity. Additionally, the social acceptability of the applications was evaluated through consultations with students, teachers, and parents.

This study focuses on the productivity of traditional and technology-supported presentations of the stepped teaching method in teaching clock- and fraction-reading/telling skills to students with mathematical learning difficulties. Participants included six students diagnosed with dyscalculia, aged 9–10 years, attending a special education school in Lefkoşa, Turkish Republic of Northern Cyprus. Students received individualized support education 3 days per week. Participants possessed basic number knowledge and could identify the hour and minute hands and recognize full hours, half hours, and quarter hours, but experienced difficulty discriminating between half hours and fractions.

The research employed a single-subject, adapted alternating treatments design to compare the two presentations of the stepped teaching method in teaching clock- and fraction-based reading/telling skills. The researcher served as the instructor, while students and classroom teachers contributed to progress monitoring. Two independent observers collected inter-observer reliability data. All sessions—including baseline, instruction, probe, generalization, and recording—were conducted individually in the special education classroom using structured data collection forms, and participants’ personal data were protected according to ethical guidelines.

The researcher administering the teaching sessions possessed the necessary professional qualifications and expertise in providing individualized support to students with mathematical learning difficulties. This expertise ensured the accurate implementation of the teaching procedures. Inter-observer reliability data were collected for both dependent and independent variables by two specialists with bachelor’s and graduate-level training, and 30% of sessions were evaluated for inter-observer and procedural reliability.

In this study, social acceptability data were collected from students, their classroom teachers, and their families. Five special education teachers who worked with the students provided their perspectives on the usability and effectiveness of clock- and fraction-based reading/telling skills using both traditional and technology-supported presentations.

The term “technology-based application” refers to a structured instructional system developed in Unity using C# programming language. The system does not employ machine learning or predictive artificial intelligence algorithms. Instead, it functions as a rule-based adaptive environment in which student responses are processed in real time through predefined conditional logic (if–then rules).

Based on participants’ accuracy and response patterns, immediate feedback is automatically provided, and subsequent instructional steps are selected within a pre-structured branching framework. These adaptations are governed by programmed decision rules rather than data-driven learning mechanisms. Accordingly, the application should be conceptualized as a technology-based instructional system that provides guided practice, systematic prompting, and immediate corrective feedback aligned with participant performance.

The adapted alternating treatments design was selected to allow systematic comparison of two instructional conditions while controlling for individual variability in learning trajectories. To reduce potential carryover effects, a minimum one-hour interval was implemented between sessions, during which participants did not engage in any structured instruction related to the target skills. This washout period was intended to reduce short-term memory interference and minimize immediate transfer effects across conditions, as recommended in single-case experimental design frameworks emphasizing control of sequence and carryover effects (Barlow et al., 2009; Gast and Ledford, 2014; Horner et al., 2005; Kratochwill et al., 2010).

Social acceptability data were also obtained from the students’ families. Two participants were siblings; therefore, five mothers, aged 29–47, provided the required information. Families shared their opinions regarding the effectiveness, practicality, and applicability of the stepped teaching method for teaching clock- and fraction-reading/telling skills in daily life. Based on these insights, all instructional sessions were conducted individually in appropriate locations within the special education center attended by the students.

The research employed a variety of tools and materials across baseline, instructional, daily probe, generalization, and recording sessions, aligned with students’ prerequisite skills. Attendance sheets and data collection forms were used to record performance in counting, reading/telling full hours, understanding part–whole relationships, discriminating full, half, and quarter-hour intervals, and comprehending simple texts. During the application sessions, the researcher utilized a tablet computer, a notebook computer, stepped teaching plans, researcher-developed instructional materials, an external hard drive, and reinforcement stimuli. Student performances were systematically recorded on data collection forms during teaching, monitoring, and generalization sessions, and all sessions were video-recorded using Google Meet.

The technology-supported application of the stepped teaching method was designed and implemented by the researcher, adapted for tablet use. The application was developed using the Unity game engine and Visual Studio C#, incorporating two- and three-dimensional scenes, as well as digital and visual content. Written texts were narrated, and visuals and videos were synchronized with technology-supported tools, integrating sound, images, and animations. In clock instruction, the clock interface was manipulated through drag-and-drop interactions, with responses processed via technology-supported narration. In fraction instruction, a pie chart model was used, allowing click and drag-and-drop interactions to be instantly calculated. For both instructional domains, student performance was supported with visual and auditory feedback to enhance learning.

## 2 Materials and methods

This study employed a single-subject, adapted alternating treatments design (Holcombe et al., 1994; Tekin-İftar, 2012; Kurt, 2012) to compare traditional and technology-supported presentations of the stepped teaching method for teaching clock- and fraction-reading/telling skills to students with dyscalculia. In this model, experimental control is established when changes observed in the dependent variable associated with one independent variable occur more rapidly and consistently than changes observed with the other independent variable (Tekin-İftar, 2012).

To reduce potential carryover effects in the adapted alternating treatments design, a one-hour interval was scheduled between instructional sessions. Temporal separation is commonly recommended in single-case research to minimize interaction between conditions, particularly when sessions are discrete and structured (Cooper et al., 2020; Gast and Ledford, 2014). Considering that the targeted skills (clock reading and fractions) are conceptually related, this interval was intended to limit immediate transfer between instructional conditions. In addition, instructional methods were systematically alternated across sessions to control for sequence effects. Taken together, these procedures were considered sufficient to reduce the likelihood of carryover effects influencing the results.

In the Turkish Republic of Northern Cyprus (TRNC), student identification for special educational needs is conducted through official referral procedures under the Ministry of National Education. In this study, the student was identified through a school-based referral due to persistent difficulties in mathematics learning. With parental consent, the student was referred to the Psychological Counseling, Guidance, and Research Unit (PDRAŞ) for formal psychoeducational assessment. The student had previously received a documented diagnosis of mathematical learning difficulties based on standardized evaluation conducted by authorized professionals, which was verified through official school records prior to participation. In addition, the researcher conducted a baseline screening of mathematics performance to ensure that the student met the prerequisite skills for the instructional tasks.

Six students were selected and interviewed, and baseline (starting-level) assessment sessions were conducted to determine the target skills, guided by the Basic Education Mathematics Curriculum (Kurt, 2012; Gast, 2010). For both instructional approaches, equivalent, functionally similar, yet independent target behaviors were identified, and the interventions were implemented in an alternating fashion, with at least 1 h between sessions to reduce carryover effects (Tekin-İftar, 2012; Tekin, 2000). Clock- and fraction-reading/telling skills were taught using a 100% correct-response criterion, and maintenance sessions were conducted on the 10th, 20th, and 35th days following the completion of instruction.

To ensure reliability, at least 30% of the sessions were evaluated for procedural fidelity and inter-observer agreement. All variables, except for the independent variables, were controlled and balanced, and consistent reinforcement stimuli were used across sessions. Instructional sessions were conducted individually in the same environment for all students. Ethical standards were maintained throughout the study, with approval obtained from the Special Education and Rehabilitation Center Directorate, and written informed consent was provided by the parents of all participants (Terzi̇oğlu and Yikmiş, 2022; Akbıyık et al., 2022).

This design allowed for a systematic comparison of traditional and technology-supported stepped teaching presentations in teaching clock- and fraction-reading/telling skills, ensuring controlled conditions, repeated measures, and rigorous reliability checks.

### 2.1 Application sessions

Following baseline assessment sessions and the establishment of stable data, instructional sessions commenced using the stepped teaching method. Clock-telling/reading and fraction-reading/telling skills were taught individually to each student. The stepped teaching method was structured along two dimensions: the horizontal dimension, which encompassed teacher presentation and student response, and the vertical dimension, which included the “do,” “show,” and “say” steps (Bachor and Freeze, 1986; Cawley and Reines, 1996; Yıkmış, 2005). In analog clock instruction, the “model” step was omitted, while maintaining equivalence between clock and fraction teaching procedures (Tekin-İftar and Kırcaali-İftar, 2012).

Instruction took place in the special education classroom during course hours, with two sessions conducted per day. For each target skill, teaching continued until three consecutive sessions produced stable performance data. Students were expected to read half-hour intervals on the analog clock with 100% accuracy. In cases of incorrect or non-responses, physical prompts were provided. Across all steps, instructional materials were utilized systematically, student responses were evaluated, and reinforcement stimuli were delivered at the conclusion of each session to support learning and motivation.

### 2.2 Traditional presentation of fraction reading/telling instruction

In this study, fraction-reading/telling skills were taught using the traditional presentation of the stepped teaching method. Following the establishment of stable baseline data during preliminary assessments, instructional sessions commenced, with the goal of having students read or verbally identify the fraction displayed on a model or picture card with 100% accuracy. Each teaching session lasted approximately 40 min and incorporated three sub-goals under each main instructional step. The instructor and student worked side by side, using sliced pie models and illustrated fraction cards. Instruction followed the “do,” “show,” and “say” sequence, in which students modeled the fraction using their own materials (“do”), demonstrated the correct fraction with the cards (“show”), and verbally identified the fraction (“say”). Student responses were systematically recorded and evaluated at each step.

Multiple examples were provided at each step. Correct responses were coded as (+), whereas incorrect or non-responses were coded as (−). If a student did not respond within 5 s, the instruction was repeated once. Persistent non-response was recorded as incorrect. If three consecutive incorrect responses occurred, physical assistance was provided on the fourth attempt. Upon successful completion of each step, the student received reinforcement before proceeding to the next step. At the end of the session, designated reinforcement was delivered to support motivation and learning.

### 2.3 Technology-supported, stepped teaching of clock and fraction

#### 2.3.1 Technology-based clock reading/telling instruction

In this study, instruction targeting half-hour intervals on the analog clock was delivered using an technology-supported stepped teaching method. Following the collection of stable baseline data during preliminary assessments, technology-assisted teaching sessions commenced.

The long-term aim (LTA) of the instruction was for students to read and verbally identify the half-hour intervals on the analog clock with 100% accuracy. Each main instructional step included four short-term aims (STA), and sessions were planned to last approximately 40 min per student, progressing according to individual performance. The instructional target included twelve half-hour intervals (1:30–12:30) on the analog clock.

#### 2.3.2 Application medium and preparation procedure

Instruction was delivered using an artificial intelligence (AI) application specifically designed for a tablet computer. The researcher and student sat side by side, with the tablet positioned to allow unobstructed viewing by the student. Prior to the sessions, students were briefly introduced to the operation of the application, and it was explained that the instruction would be game-based and interactive. A virtual instructor (“Teacher Missy”) guided the stepped teaching process, with each step reinforced by short scenarios reflecting daily-life contexts.

The application interface displayed instructional sequences such as do–do, do–show, do–say, show–do, show–show, show–say, say-do, say-show, and say-say. Student responses were evaluated in real time. Correct responses triggered visual and auditory reinforcement, whereas incorrect or non-responses produced a warning sound. If three incorrect or non-responsive attempts occurred within four trials, the researcher provided verbal or physical prompts. All responses were automatically recorded in the application’s database and concurrently logged on data collection forms to ensure accurate documentation and analysis.

#### 2.3.3 Application of instructional steps

The technology-supported stepped teaching method included nine instructional sequences, which systematically guided students from concrete to abstract representations of clock-reading/telling skills:

Do–do: when the student selected this step, the virtual teacher demonstrated the target time on the analog clock. The minute hand was placed on 6, and the hour hand was positioned between the two relevant numbers to represent the targeted half-hour. The student was then asked to replicate the same arrangement on their screen. Correct placement required the minute hand on 6 and the hour hand between the appropriate numbers. Any misplacement was recorded as an incorrect response.

Do–show: the virtual teacher generated the target half-hour on the analog clock. The student was asked to select the correct time from four clock images. Responses were evaluated using the same criteria as in the do–do step.

Do–say: the virtual teacher displayed the target time and asked the student to verbally state the clock reading. Verbal responses were checked for accuracy by both the researcher and the system.

Show–do: a picture card displaying the target half-hour was presented, and the student was asked to reproduce the same clock arrangement on the analog clock interface.

Show–show: a modeled clock card was shown, and the student was required to select the matching clock from four available images.

Show–say: the virtual teacher displayed the target clock and asked the student to verbally identify the time.

Say–do: no visual model was provided; the virtual teacher gave only a verbal directive. The student was asked to generate the corresponding time on the analog clock interface.

Say–show: the virtual teacher verbally instructed the student with the target time, and the student selected the correct clock from four picture cards.

Say–say: without any visual model or cues, the student received only a verbal instruction and was asked to correctly express the indicated time. This step was applied based on the evaluation of the student’s independent performance.

#### 2.3.4 End-of-instruction reinforcement

Upon completion of all instructional steps, students were introduced to the “Set Your Own Clock” activity available within the AI application. In this activity, students could freely manipulate the hour and minute hands of the analog clock. Each movement elicited immediate visual feedback, reinforcing previously taught skills and promoting motivation and engagement throughout the learning process.

#### 2.3.5 Technology-supported fraction reading/telling instruction

Fraction reading/telling skills were taught using the technology-supported tablet application following the collection of stable baseline data in the preliminary assessment sessions. Instruction was conducted individually for each student, with each session lasting approximately 40 min. The teaching procedures mirrored the clock-reading/telling instruction in structure and sequencing, using the stepped teaching method to guide students from concrete representations to abstract identification of fractions. Student responses were recorded and evaluated in real time, and reinforcement was provided throughout the session to maintain engagement and support learning.

#### 2.3.6 Application of instructional steps: fraction reading/telling

The technology-supported fraction instruction followed a nine-step sequence, mirroring the stepped teaching structure used in clock instruction, and gradually guided students from concrete manipulation to abstract verbal expression.

Do–do: after selecting this step, the virtual teacher first stated the total number of slices on the pie model (denominator) and the number of highlighted slices (numerator), then verbally identified the fraction (e.g., 1/2). The student was asked to count all slices, place the total on the denominator and the highlighted slices on the numerator, and read the fraction aloud. Correct responses required accurate placement and verbal reading. Correct answers triggered verbal reinforcement; incorrect or non-responses produced a warning sound. Upon completion, the student advanced to the next step.

Do–show: the virtual teacher generated the fraction model (e.g., 3/4). The student was asked to select the correct corresponding fraction from options displayed on the screen. Responses were evaluated using the same criteria as in the Do–Do step.

Do–say: the teacher displayed the fraction model and asked the student to verbally express the fraction (e.g., “two over three”). Responses were recorded by both the system and the data collection forms.

Show–do: the virtual teacher presented the target fraction on the model. The student was asked to reproduce the fraction on the screen and verbally read it.

Show–show: the teacher displayed the fraction model. The student was asked to select the matching model from multiple options.

Show–say: the virtual teacher presented the fraction visually, and the student was asked to verbally express the fraction.

Say–do: only a verbal directive was provided by the teacher (e.g., “two over three”). The student was asked to construct the fraction model on the screen and read it aloud.

Say–show: the teacher verbally expressed the target fraction. The student was asked to select the correct model based on the verbal instruction.

Say–say: no visual support was provided; the student was given a verbal instruction and asked to verbally reproduce the fraction.

#### 2.3.7 Evaluation and termination of sessions

Student responses and instructional progress were monitored according to the acceptance criteria established in the do–do step. Sessions were terminated upon completion of the Say–Say step. After each session, the virtual teacher (“Teacher Missy”) provided performance feedback and guided the student to a reinforcement activity within the application. In this activity, students could freely decorate and slice the pie model on the touchscreen, receiving immediate visual feedback. This design aimed to enhance motivation, make learning enjoyable, and provide positive reinforcement.

Student responses were systematically recorded throughout the study. In technology-supported teaching, responses were automatically logged into the database, whereas in traditional teaching, the researcher manually recorded responses in structured data forms. Correct responses were coded as (+), incorrect or no responses as (−), and verbal hints (VH) or physical prompts (PH) were additionally noted. Both instructional formats employed the same measurement and recording procedures, ensuring objectivity, consistency, and comparability. Screenshots and sample images of the technology-supported teaching are provided in the Appendix to support transparency and reproducibility.

#### 2.3.8 Daily inspection, monitoring, and generalization sessions

Throughout the study, students’ clock- and fraction-reading/telling performances were monitored through daily inspection sessions and systematically recorded. Maintenance of the acquired skills was evaluated during follow-up sessions on the 10th, 20th, and 35th days. These evaluations employed varied materials, media, and instructors to assess skill generalization. Data regarding effectiveness, productivity, and social validity were collected by the researcher, while reliability data were obtained by independent observers.

#### 2.3.9 Data on effectiveness

The effectiveness of the stepped teaching method in traditional and technology-supported formats was compared using the Adaptive Transformation Application Model. Data were collected at baseline, daily inspection, monitoring, and generalization sessions. In traditional teaching, student responses were manually recorded by the researcher, whereas in technology-supported teaching, all responses were automatically logged into the database.

Student performance was quantified by coding responses as correct (+) or incorrect (−), and the percentage of correct responses was calculated using the following formula (Equation 1):


$$\text{Correct Response Percentage}=(\frac{\text{Number of correct steps}}{\text{Total number of steps}})\times 100$$ (1)

These data were then presented graphically, and the two instructional approaches were analyzed comparatively to evaluate differences in effectiveness.

#### 2.3.10 Data on productivity

The productivity levels of traditional and technology-supported teaching were compared by considering the number of sessions required to reach the performance criterion, total sessions conducted, number of trials, number of errors, and total teaching time. In traditional teaching, these data were recorded manually on data collection forms, whereas in technology-supported teaching, the data were obtained directly from the application database and subsequently transferred to the forms. The results were tabulated and analyzed comparatively.

#### 2.3.11 Data on social availability

Social availability data were collected to evaluate the appropriateness of the teaching process, the relevance of learning objectives, and the practical applicability of the results. These data were obtained from students, parents, and special education teachers using closed-ended questionnaires. Responses were analyzed using percentage calculations.

#### 2.3.12 Data on reliability

Reliability data were collected to ensure inter-observer consistency and application fidelity. For a predetermined percentage of all sessions, both inter-observer reliability and procedural fidelity were evaluated.

Application reliability was calculated (Tekin-İftar and Kırcaali-İftar, 2012) as (Equation 2):


$$\begin{array}{l} (\text{Observed Behavior of Applicant})/\\ (\text{Planned Behavior of Applicant})\times 100 \end{array}$$ (2)

For all types of sessions, both clock and fraction teaching applications demonstrated 100% procedural fidelity and 100% inter-observer reliability.

## 3 Results

This section presents the effectiveness, productivity, and social validity results of traditional and technology-supported teaching presentations in the instruction of clock reading/telling and fraction reading/telling skills for students with dyscalculia.

### 3.1 Effectiveness results

**Figure 1:**
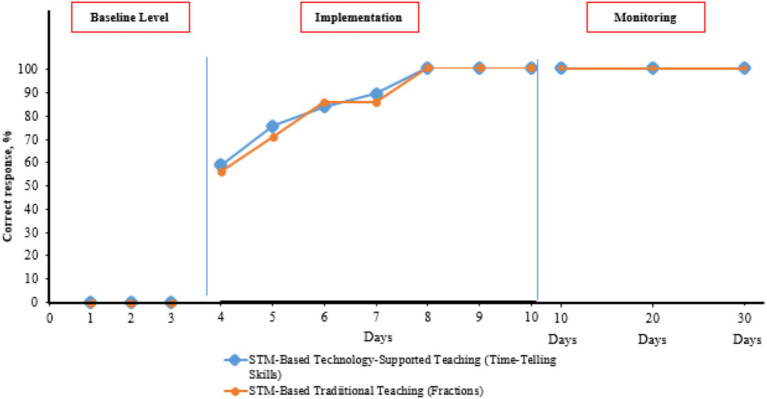
Poyraz’s correct response percentages for clock reading/telling and fraction reading/telling skills at the baseline, application, and monitoring levels.

**Figure 2:**
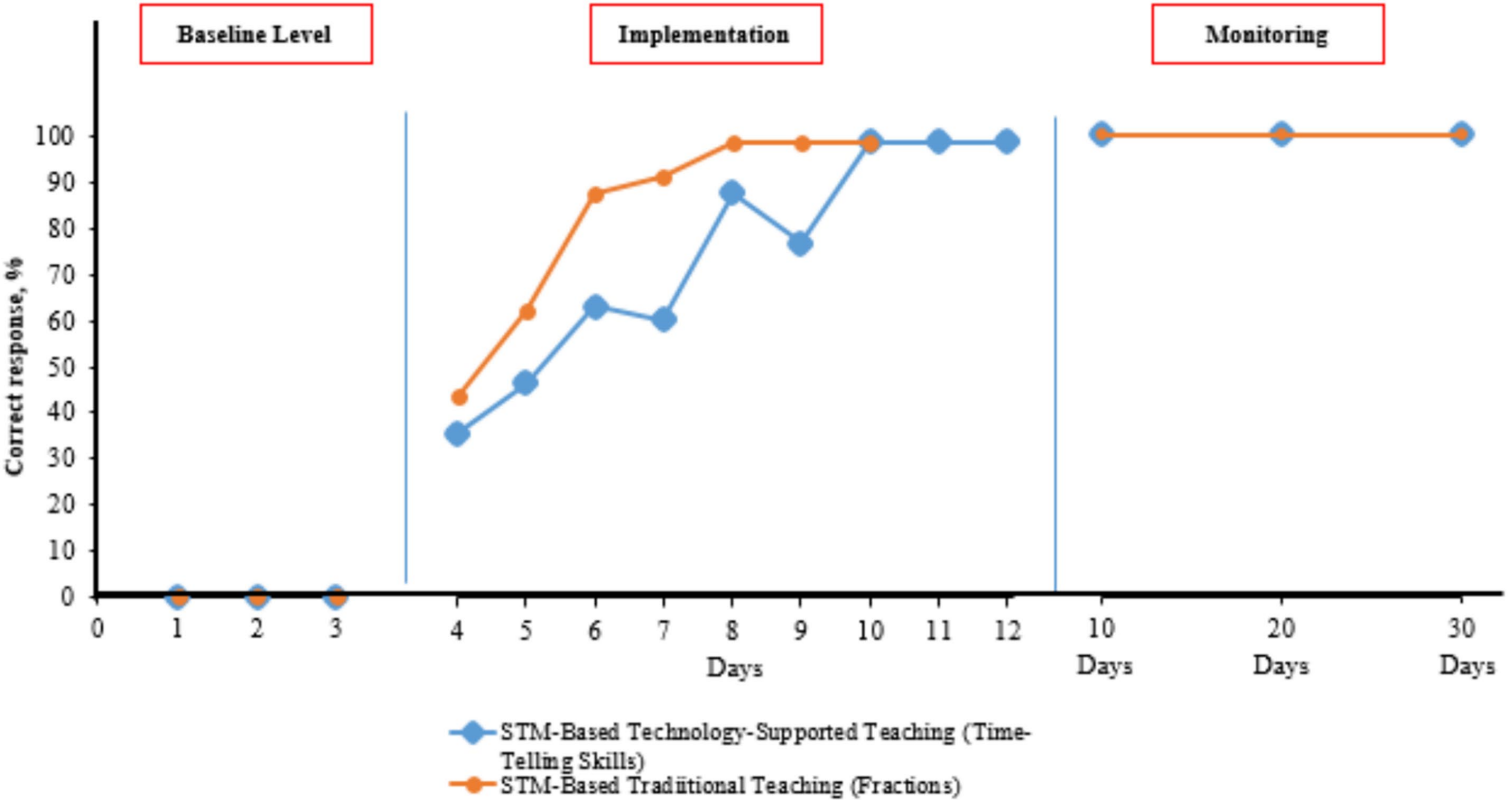
Kemal’s correct response percentages for clock teaching and fraction teaching at the starting level, application level, and monitoring level.

**Figure 3:**
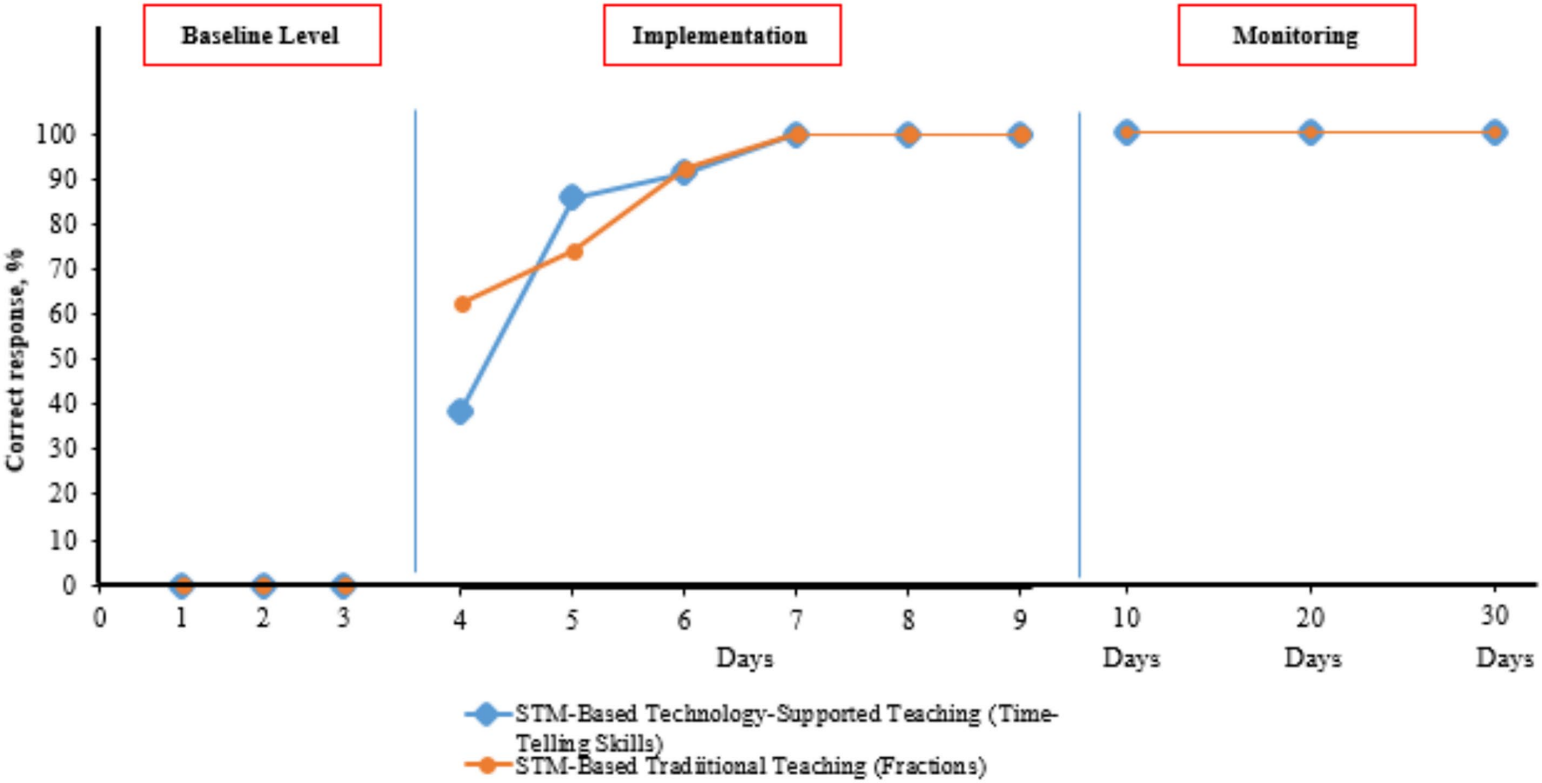
Sadem’s correct response percentages in clock reading/telling and fraction reading/telling skills were evaluated at the starting level, application level, and monitoring level.

**Figure 4:**
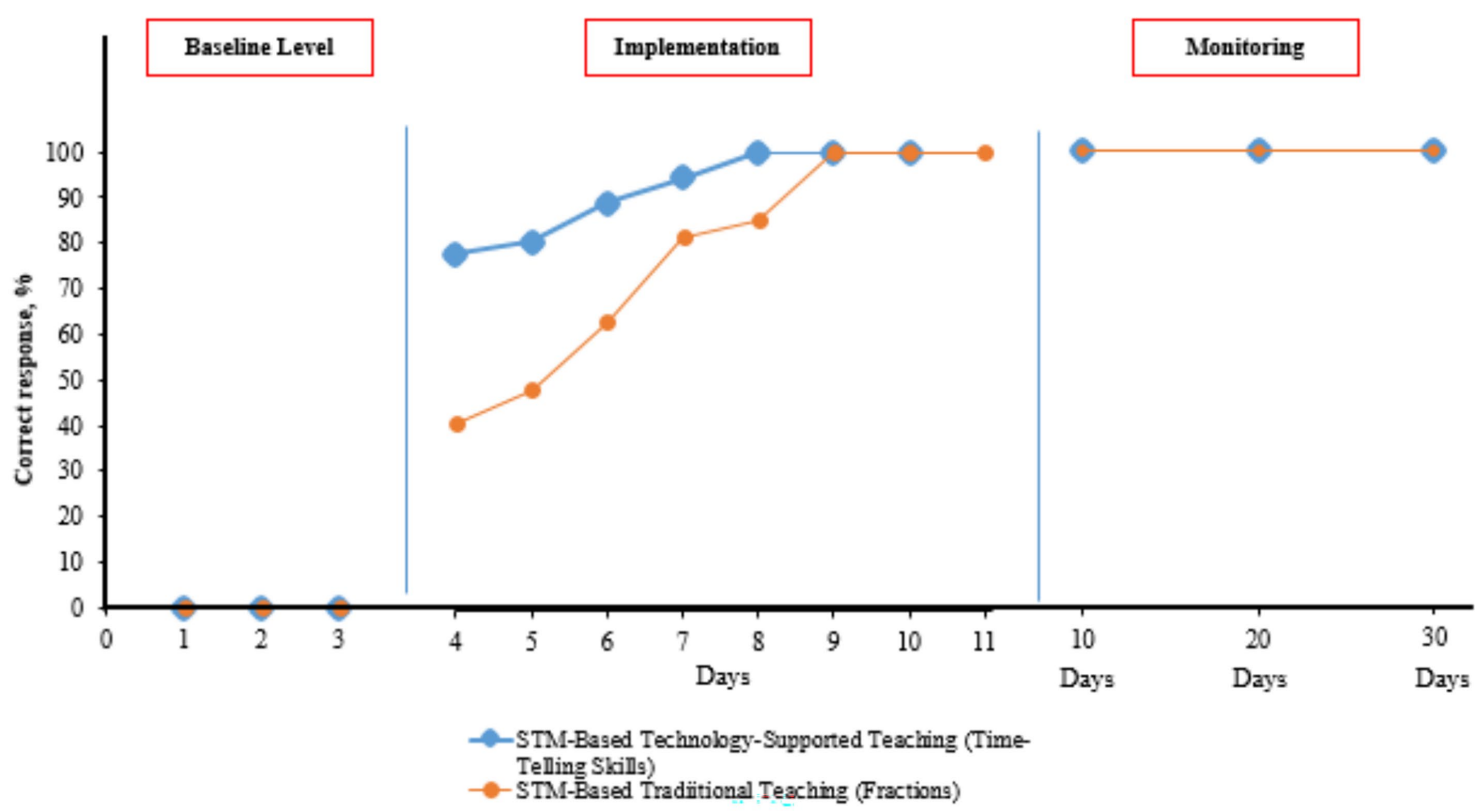
Erin’s correct response percentages in clock reading/telling and fraction reading/telling skills were examined at the starting level, application level, and monitoring level.

**Figure 5:**
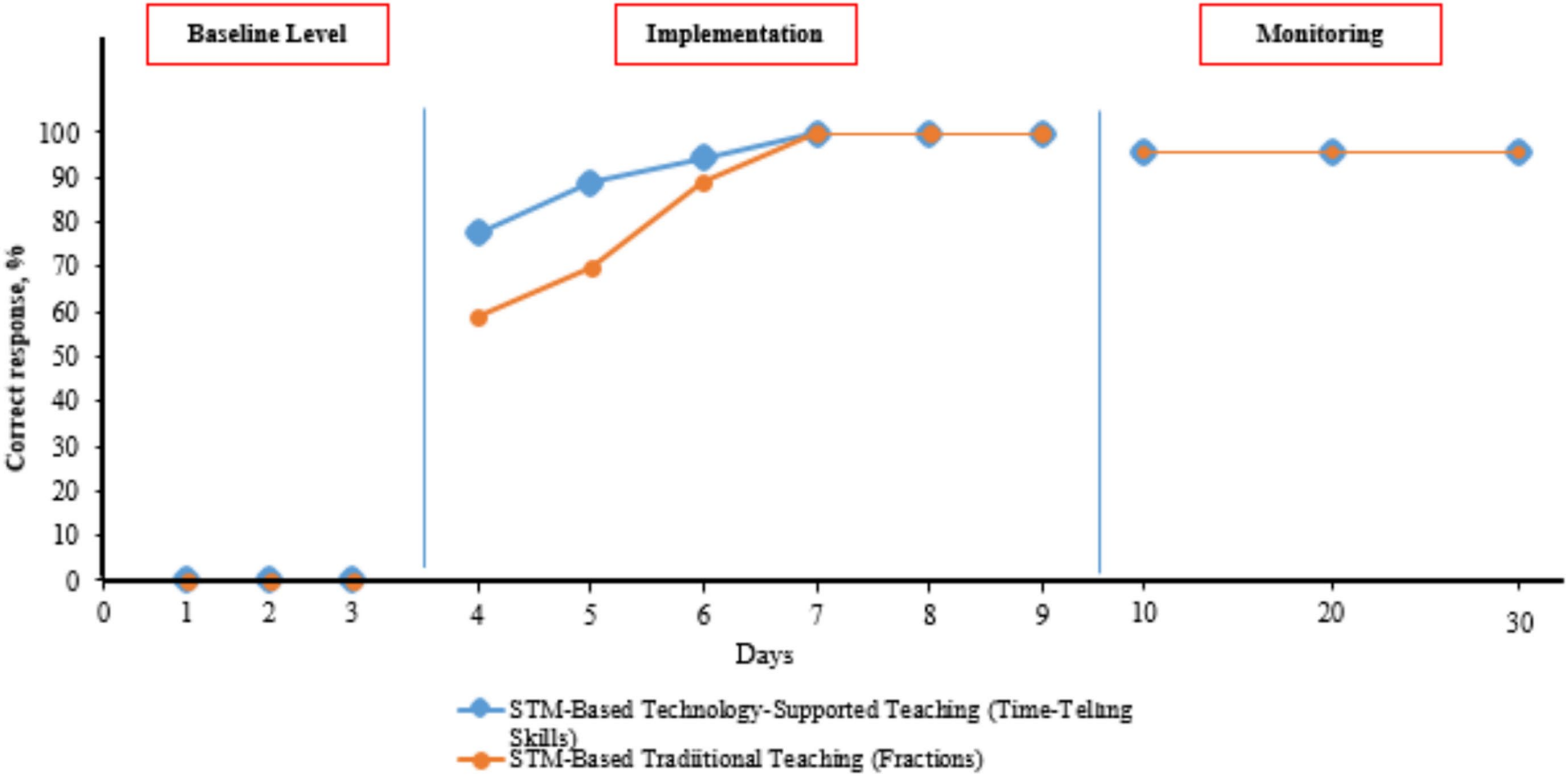
Ecrin’s correct response percentages for technology-supported clock teaching and traditional fraction teaching at the starting, application, and monitoring levels.

**Figure 6:**
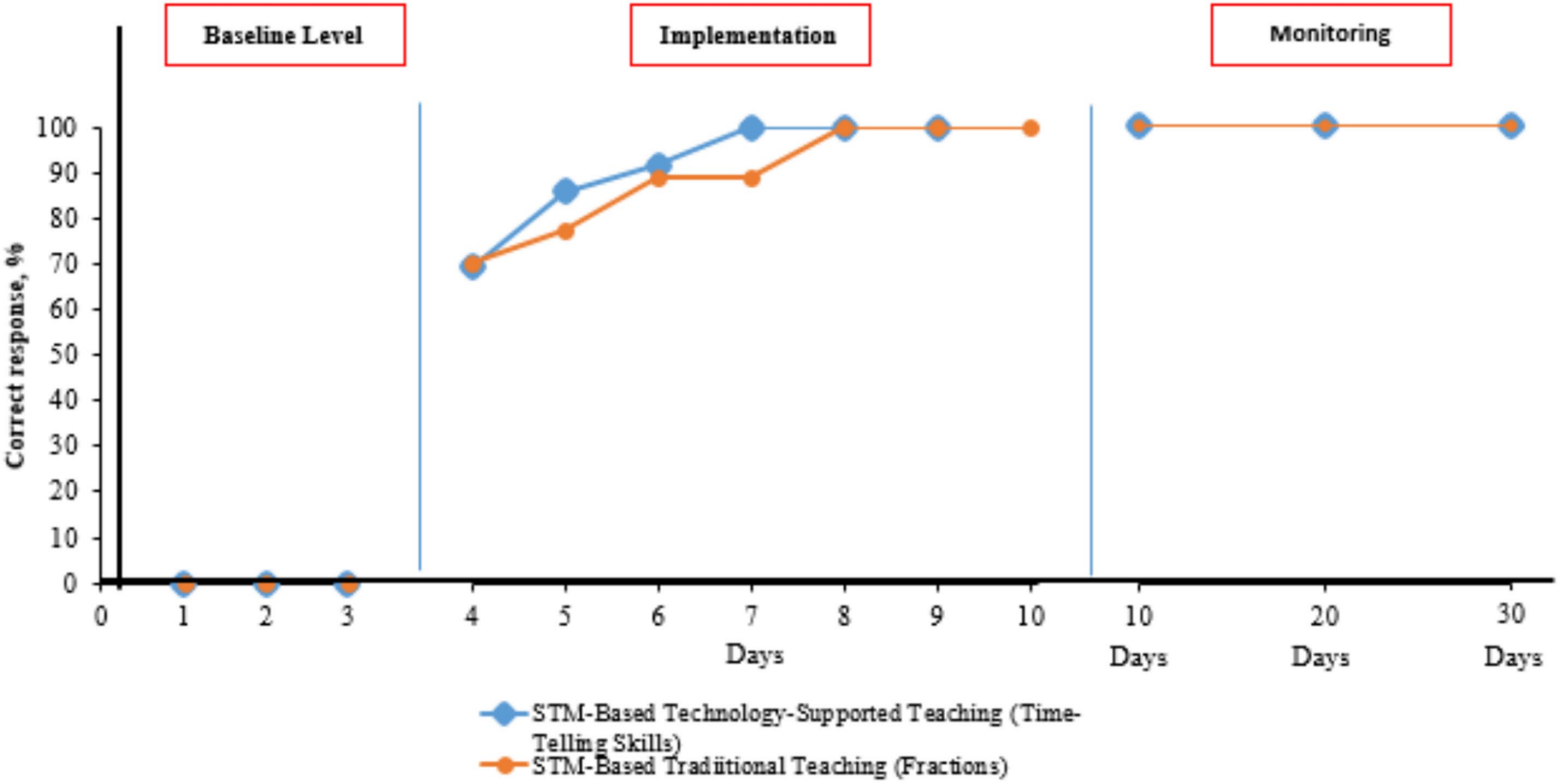
Barış’s clock and fraction skills.

**Figure 7:**
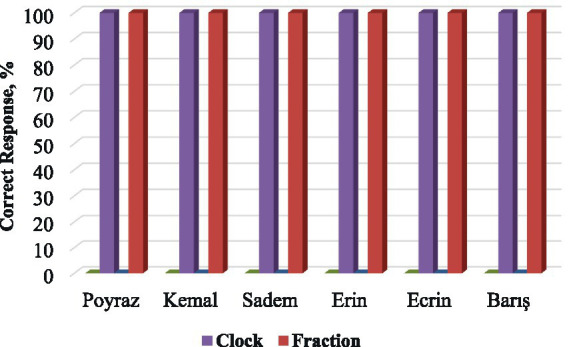
Correct response percentages of Poyraz, Kemal, Sadem, Erin, Ecrin, and Barış in clock and fraction teaching presentations.

The performances of the students Poyraz, Kemal, Sadem, Erin, Ecrin, and Barış in clock reading/telling and fraction reading/telling skills are illustrated in Figures 1–6, with generalization data presented in Figure 7.

For each student, effectiveness data are displayed using line graphs, while generalization data are presented using column graphs. The line graphs depict baseline data along with application and monitoring session results, whereas the column graphs show pre-test and post-test results for generalization. Baseline data were obtained from pre-instruction and inspection sessions. Application data were collected during daily inspection sessions conducted after each teaching session. Monitoring data were recorded during follow-up sessions on the 10th, 20th, and 35th days post-intervention.

The following sections provide detailed performance data for each student individually.

#### 3.1.1 Effectiveness data about Poyraz

The figure illustrates performance in technology-supported clock teaching and traditional fraction teaching applications across the three phases.

At the baseline, Poyraz’s performance in both clock and fraction reading/telling skills was 0%.

During the application phase, both teaching presentations achieved the criterion level. Poyraz demonstrated 100% correct responses in the fifth, sixth, and seventh consecutive sessions in both teaching models.

Follow-up monitoring data indicated that these skills were maintained at 100% accuracy on the 10th, 20th, and 35th days after the completion of instruction, demonstrating successful retention of the taught skills.

#### 3.1.2 Effectiveness data about Kemal

Clock teaching (technology-based) and fraction teaching (traditional) correct response percentages were recorded at the starting, application, and monitoring stages.

At the starting level, Kemal’s clock reading/telling and fraction reading/telling performances were approximately 0%. At the application level, the fraction teaching reached the criterion level by the fifth session, with Kemal demonstrating 100% accuracy in the fifth, sixth, and seventh sessions consecutively. For clock teaching (technology-supported), the criterion was reached after the seventh session, and 100% accuracy was observed in the seventh, eighth, and ninth sessions consecutively.

Monitoring data show that both skills were retained at 100% accuracy during the follow-up sessions on the 10th, 20th, and 35th days after the conclusion of instruction.

It is important to note that all participants were selected based on a diagnosis of dyscalculia. Additionally, Kemal exhibited signs of attention deficit and hyperactivity during the application sessions, which were especially evident at the starting and teaching stages. Although ADHD was not a formal co-diagnosis in the preliminary criteria, these behaviors impacted his ability to maintain attention and follow directives. Consequently, Kemal required more sessions and additional time to reach the criterion, which may be attributed to these attention-related challenges.

These findings highlight that, alongside mathematical learning difficulties, individual behavioral characteristics such as attention regulation and self-management can influence both the progress and productivity of the teaching process. Kemal’s data therefore underscore the importance of considering personal differences and accompanying behavioral traits in designing and implementing instruction.

#### 3.1.3 Effectiveness data about Sadem

Figure 3 illustrates her performance across technology-supported clock teaching and traditional fraction teaching.

At the starting level, Sadem’s performance in both skills was around 0%, indicating no prior mastery of the targeted behaviors. During the application phase, both teaching presentations demonstrated systematic improvement. Sadem reached the criterion level for both technology-supported clock teaching and traditional fraction teaching after the fourth session. She maintained 100% accuracy successively in the fifth, sixth, and seventh sessions.

Monitoring data indicated that the acquired skills were sustained over time. Assessments conducted on the 10th, 20th, and 35th days following the completion of instruction showed that Sadem retained 100% accuracy in both clock and fraction reading/telling skills.

These findings suggest that both traditional and technology-supported stepped teaching methods were effective for Sadem, with technology-supported instruction providing rapid skill acquisition and durable learning outcomes.

#### 3.1.4 Effectiveness data about Erin

Figure 4 illustrates her performance across technology-supported clock teaching and traditional fraction teaching.

At the starting level, Erin’s performance for both skills was approximately 0%, indicating that she had not yet acquired the targeted behaviors. During the application phase, a systematic improvement was observed in both teaching presentations. Erin gradually reached the criterion level in successive sessions, demonstrating increasing accuracy in both technology-supported clock teaching and traditional fraction teaching.

Monitoring data revealed that the acquired skills were maintained over time. Assessments conducted on the 10th, 20th, and 35th days following the completion of instruction showed sustained correct response percentages, indicating effective retention of both clock and fraction reading/telling skills.

These findings suggest that the stepped teaching methods—both traditional and technology-supported—were effective for Erin, promoting both skill acquisition and long-term retention.

At the application level, Erin’s performance in both clock and fraction teaching skills increased steadily. She reached the criterion for technology-supported clock teaching at the fifth session, while the criterion for traditional fraction teaching was achieved at the sixth session. Erin demonstrated 100% accuracy consecutively in the technology -supported teaching during the fifth, sixth, and seventh sessions, and in the traditional teaching during the sixth, seventh, and eighth sessions.

Monitoring data indicated that both skills were retained at 100% accuracy during follow-up sessions on the 10th, 20th, and 35th days after the completion of instruction.

These results indicate that both technology-supported and traditional stepped teaching presentations were effective for Erin in acquiring and maintaining clock reading/telling and fraction reading/telling skills.

#### 3.1.5 Effectiveness data about Ecrin

At the starting level, Ecrin’s performance was approximately 0% for both skills.

During the application phase, both skills showed a marked improvement. Ecrin reached the criterion level for both technology -supported and traditional teaching presentations by the fourth session. She demonstrated 100% accuracy consecutively in the fourth, fifth, and sixth sessions for both teaching methods.

At the monitoring level, Ecrin maintained 100% accuracy in both clock and fraction skills, as evidenced by the assessment sessions conducted on the 10th, 20th, and 35th days following the completion of teaching. These findings indicate that Ecrin benefited effectively from both technology-supported clock teaching and traditional fraction teaching presentations, demonstrating sustained learning and skill retention over time.

#### 3.1.6 Effectiveness data about Barış

At the starting level, Barış demonstrated 0% accuracy in both clock and fraction skills. During the application phase, his performance improved steadily in both technology-supported clock teaching and traditional fraction teaching, reaching the criterion level of 100% precision. Specifically, in the traditional fraction teaching presentation, Barış reached the criterion at the fifth session and maintained 100% accuracy consecutively in the fifth, sixth, and seventh sessions. In the technology-supported clock teaching, he reached the criterion at the fourth session and achieved 100% accuracy consecutively in the fourth, fifth, and sixth sessions. Monitoring sessions conducted on the 10th, 20th, and 35th days after the completion of teaching confirmed that Barış retained 100% precision in both skills. These results indicate that both technology -supported and traditional teaching presentations were highly effective for Barış in acquiring and maintaining clock and fraction skills.

#### 3.1.7 Generalization of the stepped teaching method in traditional and technology-supported presentations for clock and fraction reading/telling skills

Figure 7 presents the generalization pre-test and post-test findings for correct responses from Poyraz, Sadem, Kemal, Erin, Ecrin, and Barış in clock and fraction reading/telling skills. As shown in Figure 7, at the pre-test stage, none of the students demonstrated correct responses for either skill. However, following the intervention, all students were able to generalize the taught skills to different individuals and equipment, achieving 100% accuracy in the post-test. These results indicate that both traditional and technology-supported stepped teaching presentations were effective in supporting not only the acquisition but also the generalization of clock and fraction skills.

#### 3.1.8 Productivity results

Table 1 presents the productivity data for six students in terms of number of sessions, number of trials, number of errors, and total teaching time until reaching the criterion in clock and fraction teaching. Findings indicate that both traditional and technology -supported presentations required a similar number of sessions and trials to reach 100% accuracy, with all students showing 0% errors, reflecting a controlled and consistent learning process.

**Table 1:** Productivity results about technology-supported teaching and traditional teaching presentations of stepped teaching method.

Pupil	Independent variable	Dependent variable	Number of sessions	Number of tries	Number of errors	Total time of teaching
Poyraz	Traditional teaching of stepped teaching method	Time telling/reading	7	108	0	4 h 3 min
	*Technology-supported teaching* of stepped teaching method	Fraction reading/telling	7	108	0	2 h 42 min
Kemal	Traditional teaching of stepped teaching method	Time telling/reading	7	108	0	3 h 47 min
	*Technology-supported teaching* of stepped teaching method	Fraction reading/telling	9	144	0	3 h 27 min
Sadem	Traditional teaching of stepped teaching method	Time telling/reading	6	72	0	3 h 38 min
	*Technology-supported teaching* of stepped teaching method	Fraction reading/telling	6	72	0	2 h 5 min
Ecrin	Traditional teaching of stepped teaching method	Time telling/reading	6	72	0	3 h 30 min
	*Technology-supported teaching* of stepped teaching method	Fraction reading/telling	6	72	0	2 h
Erin	Traditional teaching of stepped teaching method	Time telling/reading	8	126	0	4 h 30 min
	*Technology-supported teaching* of stepped teaching method	Fraction reading/telling	7	108	0	2 h 20 min
Barış	Traditional teaching of stepped teaching method	Time telling/reading	7	108	0	3 h 50 min
	*Technology-supported teaching* of stepped teaching method	Fraction reading/telling	6	72	0	2 h

In terms of total teaching time, technology-supported presentations were more efficient for most students. For example:

- Poyraz: technology-supported 2 h 42 min vs. Traditional 4 h 3 min
- Sadem: technology-supported 2 h 5 min vs. Traditional 3 h 38 min
- Ecrin: technology-supported 2 h vs. Traditional 3 h 30 min
- Erin: technology-supported 2 h 20 min vs. Traditional 4 h 30 min
- Barış: technology-supported 2 h vs. Traditional 3 h 50 min

Kemal showed a slightly higher number of sessions and trials in the technology-supported teaching (9 sessions, 144 trials), yet total teaching time was still shorter than traditional teaching (Technology-supported 3 h 27 min vs. Traditional 3 h 47 min).

Overall, while both teaching presentations were similarly effective in reaching the criterion, technology-supported teaching demonstrated a clear advantage in time productivity, highlighting its potential to provide a more efficient learning process.

#### 3.1.9 Social validity results

Social validity data were collected from students, families, and teachers using closed-ended forms to determine stakeholders’ perceptions regarding the meaningfulness of the results, the suitability of the teaching methods employed, and the importance of the research aims. The findings indicate that all three stakeholder groups evaluated the application positively.

Procedural fidelity was assessed using a checklist of instructional steps, with a trained observer recording whether each step was implemented as planned during selected sessions. Fidelity was determined based on the proportion of correctly implemented steps.

Following the research, the Social Validity Form for Students revealed that all students considered clock and fraction reading skills important. They reported that these skills were instrumental for their academic and daily life activities, and they observed significant improvement in their performance after the teaching period. Furthermore, students expressed satisfaction with the technology -supported teaching presentation and indicated that they applied the skills they acquired in their homework and daily routines. These findings suggest that the teaching process was both acceptable and instrumental from the students’ perspective.

Similarly, social validity results from families were highly positive. All participating families reported that clock and fraction reading skills were important for their children and contributed to academic, daily, and independent life areas. Families also observed notable improvements in their children’s performance and expressed satisfaction with both the traditional and technology-supported teaching methods. Additionally, families indicated a desire to implement the teaching approach in the home environment, highlighting the practical and applicable nature of the method.

Teachers’ evaluations also reflected high social validity. All teachers responded positively to the items in the social validity form. They emphasized that clock and fraction reading skills were important for students, that the teaching process was efficient and applicable, and that significant progress was observed among the students. Teachers further noted that students transferred the acquired skills to in-class activities, homework, and daily life routines.

Overall, the stepped teaching method, implemented in both traditional and technology-supported formats, was widely accepted and considered effective, meaningful, and applicable by students, families, and teachers alike.

## 4 Discussion

This research aimed to compare traditional and technology-supported presentations of the stepped teaching method for teaching fraction reading/telling and clock reading/telling skills, examining both effectiveness and productivity. In addition, social validity perceptions of students, families, and teachers were investigated. Both traditional and technology-supported presentations were found to be highly effective in the acquisition, maintenance, and generalization of targeted skills, with no significant differences in overall effectiveness. These findings align with previous researches on direct and stepped teaching methods, which emphasize their efficacy in teaching complex mathematical skills (Dağseven, 2001; Horn et al., 2006; Flores and Kaylor, 2007; Atmaca and Girli, 2020; Karabulut and Yıkmış, 2010).

Productivity analyses indicated that technology -supported presentations offered advantages in terms of time efficiency. Some students required fewer sessions and shorter total teaching times to reach criterion levels, consistent with literature showing that technology-supported interventions can accelerate learning and reduce errors (Benavides-Varela et al., 2020; Aspiranti and Larwin, 2021). This suggests that integrating technology-based supports into stepped teaching may contribute to faster acquisition without compromising accuracy.

Social validity results were highly positive across all stakeholders. Students reported increased motivation and engagement, families observed improvements in academic and daily-life applications, and teachers highlighted efficiency and applicability. These findings reflect the motivational and practical benefits of technology-enhanced learning and support the use of technology-supported teaching as an acceptable and functional educational tool (Acungil, 2014; Svane et al., 2023; Ciampa, 2012).

One noteworthy observation in the present study was that one participant exhibited characteristics associated with Attention-Deficit/Hyperactivity Disorder (ADHD), which may have influenced interaction with the instructional conditions. Prior research suggests that digital learning environments can both support and challenge learners with ADHD. On the one hand, features such as interactivity, immediate feedback, and adaptive pacing may enhance engagement and sustain attention (DuPaul and Stoner, 2014; Shaw et al., 2014). On the other hand, highly dynamic or visually rich interfaces may lead to overstimulation, potentially increasing distractibility and cognitive load in some learners (Mayer, 2014; Sweller, 2011).

In this context, it is possible that the technology-based instructional tool used in the present study may have differentially affected performance depending on the learner’s attentional profile. While such tools may offer advantages for maintaining engagement, they may also introduce competing stimuli that interfere with sustained focus. Therefore, these findings highlight the importance of carefully balancing interactivity and simplicity when designing digital learning tools for students with attention-related difficulties. Future research should further investigate how specific design features of technology -supported instruction influence learning outcomes for individuals with ADHD.

Observational data further highlighted that visual, verbal, and story-based elements in technology-supported presentations increased attention and engagement. Tablet-based interactions allowed students to navigate tasks independently, and instant feedback reinforced learning. However, students with attention deficit and hyperactivity disorder (ADHD) faced challenges, particularly in the “say” step, where verbal expression and precision were required. This suggests that technology-supported teaching must include structured, concise directives and visual cues for students with attentional difficulties, while traditional teaching may offer advantages through teacher-guided attention management (Alloway and Alloway, 2015; Christakis, 2020; DuPaul and Jimerson, 2014).

The systematic structure of the stepped teaching method proved critical across both presentations. The “do–show–say” steps scaffolded learning from simple to complex, reduced errors, and supported long-term skill maintenance. Monitoring sessions at the 10th, 20th, and 35th days confirmed that skills were retained at 100% accuracy, emphasizing the method’s effectiveness in both acquisition and maintenance (Tekin-İftar and Kırcaali-İftar, 2012; Engelmann and Carnine, 1982). Technology-supported presentations enhanced motivation and reduced teaching time once initial preparation was complete, while repeated and structured steps reinforced learning (Rosenshine, 2012; Cooper et al., 2020).

This study provides a unique contribution to the literature by comparatively evaluating traditional and technology-supported presentations of the stepped teaching method for students with mathematical learning deficits. It demonstrates that technology-supported presentations can complement systematic teaching methods, increasing motivation and time efficiency while maintaining effectiveness. The findings suggest that integrating technology with structured teaching strategies is a promising approach for supporting students with dyscalculia, although attention and behavioral considerations must be addressed to optimize learning outcomes.

Both traditional and technology-supported stepped teaching methods are effective, acceptable, and functionally applicable. Technology-supported teaching offers additional benefits in motivation and time efficiency, while traditional teaching provides flexible support for students with attentional or directive-following challenges. These results highlight the potential of combining systematic teaching methods with technology to create engaging, effective, and sustainable learning experiences for students with mathematical learning deficits.

## 5 Conclusion

This study examined traditional and technology-supported presentations of the stepped teaching method for teaching clock and fraction reading/telling skills to students with dyscalculia. The findings indicate that both instructional formats were associated with improvements in participants’ performance. All students reached the criterion levels, and maintenance and generalization were observed in follow-up sessions.

Although no clear differences in overall effectiveness were found between the two instructional formats, productivity outcomes suggested that the technology-supported presentation was associated with reduced completion time and fewer errors in some cases. However, because the instructional conditions were not fully balanced across identical target skills, these comparative findings should be interpreted cautiously.

Social validity results indicated that both approaches were perceived as acceptable and functional by students, families, and teachers, suggesting that structured instructional procedures can be implemented in real educational contexts with positive acceptance.

Overall, the stepped teaching method appears to be a promising approach for supporting mathematical skill acquisition in students with learning difficulties. However, conclusions regarding the relative advantages of technology-supported should be considered preliminary due to methodological limitations, including the small sample size and the lack of fully controlled comparisons.

Future research should employ more rigorous experimental controls, larger samples, and clearly defined technology-supported or technology-based instructional components to better evaluate comparative effectiveness across equivalent skills and settings.

### 5.1 Implications of the study

This study suggests that the stepped teaching method supported the acquisition of targeted mathematical skills in participating students across both traditional and technology-supported formats. However, comparative interpretations should be made with caution, as instructional conditions were not fully balanced across identical target skills. Thus, no definitive conclusions can be drawn regarding the relative effectiveness of the two approaches. Although the technology-supported format appeared to reduce instructional time in some cases, this finding remains preliminary.

The results underscore the importance of individualized instruction. While technology-supported delivery may enhance engagement and independent responding, traditional instruction may provide more flexible support for students requiring additional guidance. Social validity findings indicated that both approaches were perceived as acceptable and feasible.

In addition, the positive social validity findings indicate that technology-supported instruction is practical, acceptable, and applicable in real-life contexts. This supports its integration into educational settings.

Overall, the study suggests that combining systematic teaching methods with technology is a promising approach for improving both the efficiency and quality of mathematics instruction for students with dyscalculia.

### 5.2 Limitations and recommendations for future research

This study has several limitations. First, the small sample size, consistent with single-case designs, limits the generalizability of the findings. More importantly, the instructional conditions were not fully balanced across identical target skills, which restricts the validity of direct comparisons between traditional and technology-supported formats. Therefore, any differences observed between conditions should be interpreted with caution.

The intervention required specific technological resources and was implemented in a one-to-one format with a limited follow-up period, which may affect feasibility and the interpretation of maintenance effects. Furthermore, individual differences (e.g., attention-related characteristics) were observed but not systematically examined.

Future research should employ larger samples and more rigorously controlled designs with balanced implementation across equivalent skills. Clearer operationalization of technology-supported or technology-based components is also needed. Additionally, studies examining a broader range of skills, longer follow-up periods, and different instructional formats (e.g., group-based or hybrid models) would strengthen the evidence base.

## Data Availability

The original contributions presented in the study are included in the article/supplementary material, further inquiries can be directed to the corresponding author.
